# Unicompartmental knee arthroplasty combined with posterior cruciate ligament reconstruction: a case report

**DOI:** 10.1186/s12891-024-07492-0

**Published:** 2024-05-11

**Authors:** Tong Zheng, Longzhuo Du, Ziyue Chu, Lei Li, Binglong Li, Baoqing Zhang, Xuezhou Li, Peilai Liu, Qunshan Lu

**Affiliations:** 1 Department of Orthopaedics, Qilu Hospital of Shandong University, Jinan, 250102 China; 2 Cheeloo College of Medicine, Shandong University, Jinan, 250102 China

**Keywords:** Unicompartmental knee arthroplasty, Posterior cruciate ligament reconstruction, Knee osteoarthritis, Case report

## Abstract

**Background:**

In this study, we present the unique case of a patient with knee osteoarthritis (OA) of the medial compartment and posterior cruciate ligament (PCL) deficiency who underwent simultaneous medial unicompartmental knee arthroplasty (UKA) and PCL reconstruction.

**Case presentation:**

A 49-year-old male patient presented with a 1-year history of pain and instability in the left knee. The patient had previously experienced a trauma-related injury to the PCL of the left knee that was left untreated. Imaging and physical examination confirmed the presence of left medial knee OA along with PCL rupture. To address these issues, the patient underwent UKA combined with PCL reconstruction. The patient’s Lysholm score was 47 before surgery and 81 three months after surgery, the Western Ontario and McMaster Universities Osteoarthritis Index (WOMAC) score was 29 before surgery and 18 three months after surgery, and the International Knee Documentation Committee (IKDC) subjective score was 56.3 before surgery and 74.7 three months after surgery. Six months after surgery, the patient's gait returned to normal, and he was able to jog.

**Conclusion:**

This case report presents the first instance of UKA combined with PCL reconstruction and introduces a novel treatment approach for patients suffering from medial knee OA and ligament injury.

## Background

Unicompartmental knee arthroplasty (UKA) is an effective treatment option for unicompartmental knee osteoarthritis (OA). This procedure offers several advantages, including reduced trauma, faster recovery, and improved functionality compared to total knee arthroplasty (TKA) [1]. UKA has also been shown to be effective in various difficult scenarios, including reoperation following high tibial osteotomy (HTO) failure and UKA combined with anterior cruciate ligament reconstruction (ACLR) for treating unicompartmental OA with compromised anterior cruciate ligament function, all of which have demonstrated satisfactory outcomes [2, 3]. The indication for traditional UKA is intact ligament function [4]. Studies have shown that for patients with unicompartmental OA of the knee and anterior cruciate ligament (ACL) injury, combining UKA with ACL reconstruction (ACLR) can yield satisfactory outcomes [5, 6]. These good results achieved by ACLR combined with UKA suggest that posterior cruciate ligament reconstruction (PCLR) can also be used to provide a stable knee joint structure for UKA in patients who suffer from OA caused by an intact posterior cruciate ligament (PCL). However, we did not find any reported cases related to the combination of medial UKA and PCLR in our investigation. In this study, we are the first to report the case of a patient with medial knee OA and PCL deficiency who underwent medial UKA combined with PCLR simultaneously.

## Case presentation

A 49-year-old man presented with a 1-year history of left knee pain with a limited range of movement. The patient fell on his left knee 20 years prior, and a PCL injury was suspected but was not treated. During the physical examination, a varus deformity was observed in the patient's left knee joint. Anteromedial tenderness of the left knee was evident, and the range of motion (ROM) was 0–130°. The posterior drawer test and the Lachman test were positive, while the front drawer test was negative, and no laxity of the collateral ligament was found. The patient, whose body mass index (BMI) was 22.5, did not have any chronic diseases, such as hypertension or coronary heart disease, nor did he have any endocrine or metabolic disorders, such as diabetes or gout.

**Fig. 1:**
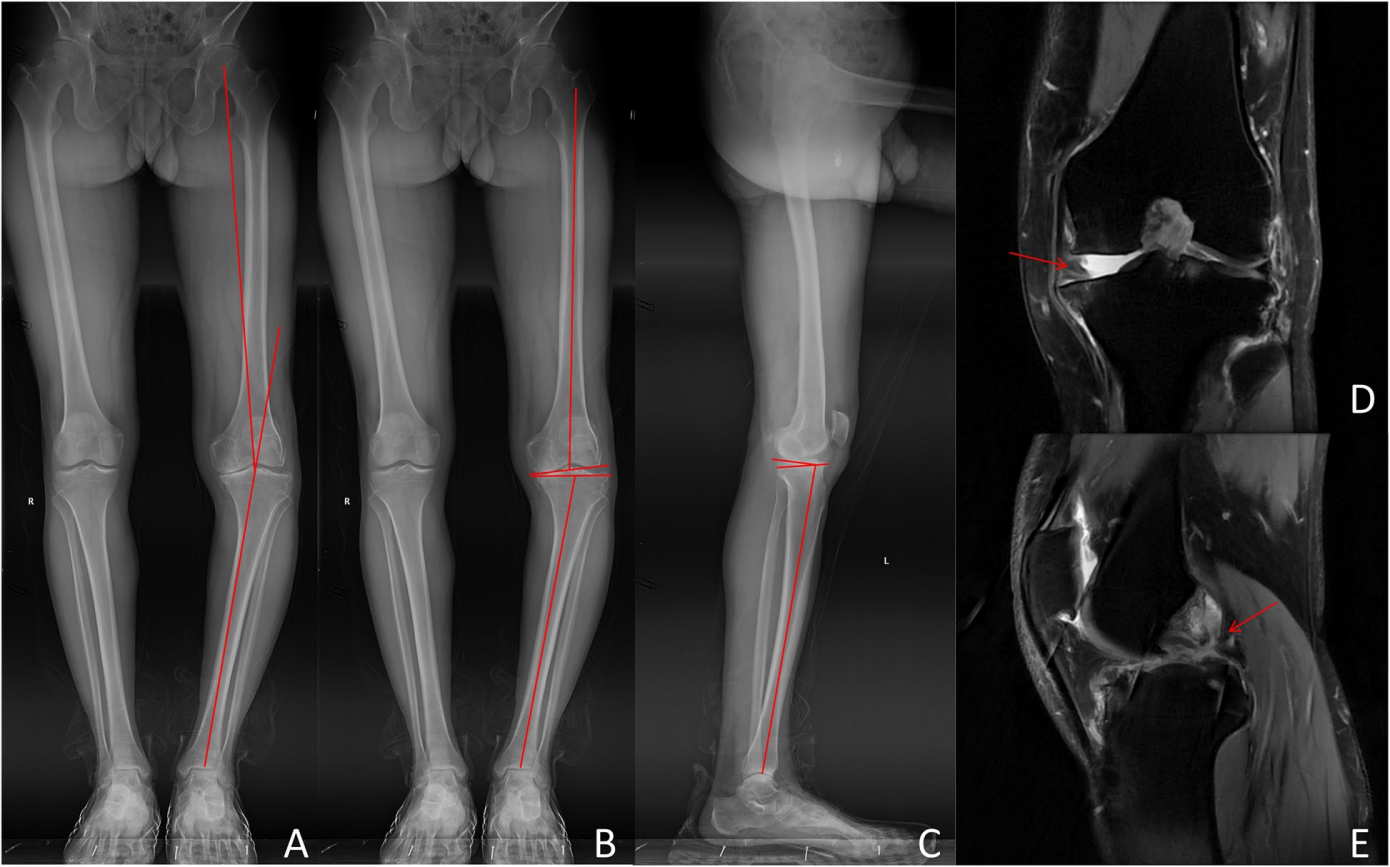
Preoperative imaging data of the patient. The hip-knee-ankle angle was 13.6° (**A**), the lateral distal femoral angle was 83.4° (**B**), the medial proximal tibial angle was 80.5° (**B**), the posterior inclination of the tibial plateau was 11.5°, and the joint line convergence angle was 6.0° (**C**). MRI revealed complete stripping of the medial cartilage (**D**) and rupture of the posterior cruciate ligament (**E**).

Anteroposterior and lateral X-rays of the patient’s left knee revealed a narrow joint space in the medial compartment and a normal joint space in the lateral compartment. The Kellgren-Lawrence grade was class IV. A patellar axial X-ray showed that the patellofemoral joint space was normal. Postoperative full-length anteroposterior standing X-rays clearly demonstrated a varus deformity of the patient's left knee, with a measured hip-knee-ankle angle of 13.6° in varus (Fig. 1A). The lateral distal femoral angle was 83.4° (Fig. 1B), the medial proximal tibial angle was 80.5°, the joint line convergence angle was 6.0° (Fig. 1B), and the posterior tibial slope was 11.5° (Fig. 1C). MRI revealed complete stripping of the medial cartilage in the left knee joint, partial tearing of the ACL with intact continuity, and the absence of continuity in the PCL (Fig. 1D and E). The patient's blood and cardiopulmonary function tests showed no important abnormalities. The preoperative Lysholm score was 47, the Western Ontario and McMaster Universities Osteoarthritis Index (WOMAC) score was 29, and the International Knee Documentation Committee (IKDC) subjective score was 56.3.

**Fig. 2:**
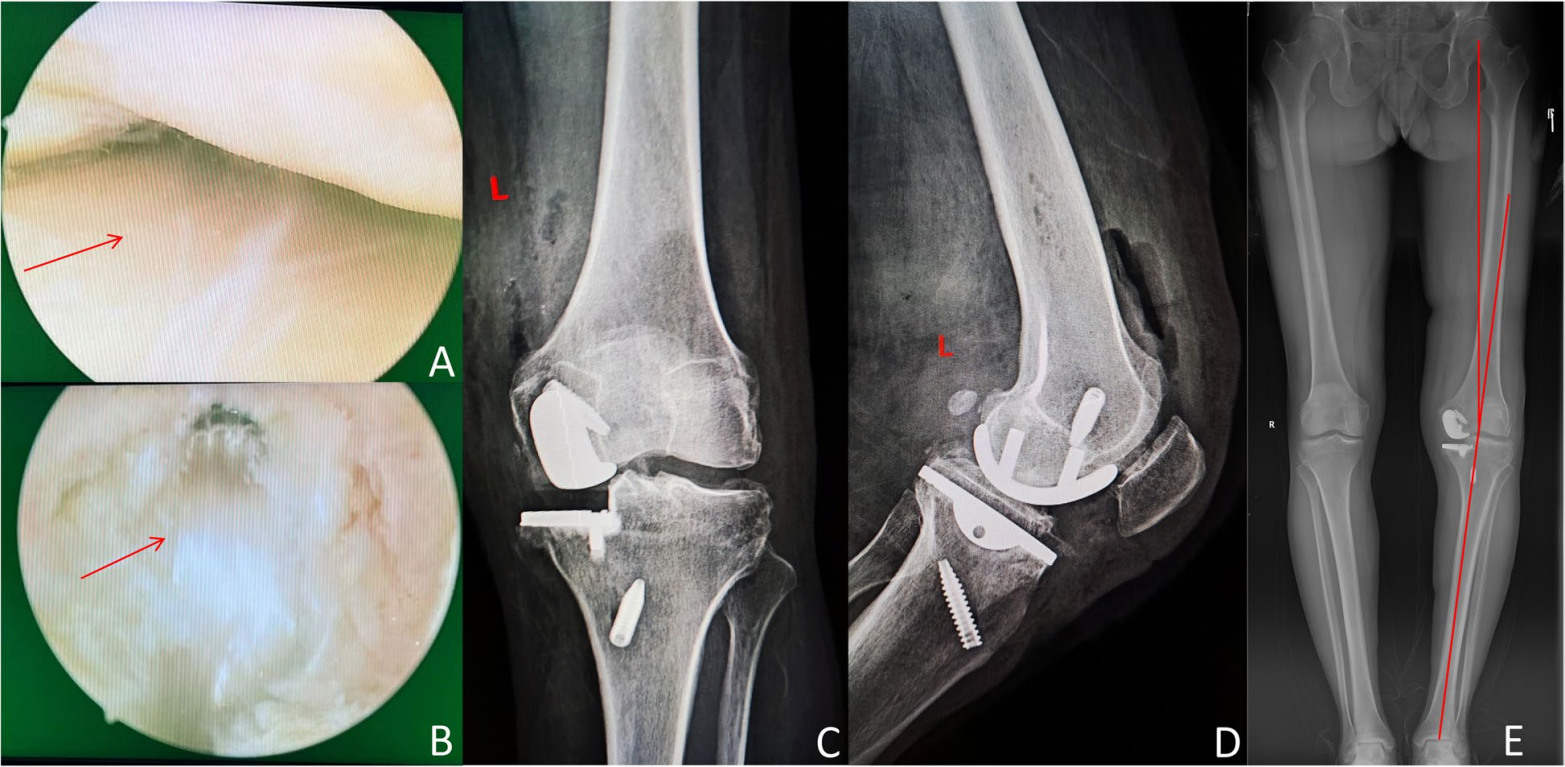
Intraoperative situation and postoperative X-ray. Under arthroscopy, the lateral cartilage was intact (**A**), and the reconstructed PCL was in the proper position (**B**). Postoperative X-ray showed that the position of the implant was ideal, and the varus deformity was substantially corrected (**C**, **D** and **E**)

PCLR and UKA were decided to perform simultaneously in order to comprehensively address the patient's condition. Following general anaesthesia, standard anterolateral portals were established as a routine procedure after a tourniquet was applied. Exploration revealed complete stripping of the medial cartilage surface, while no apparent damage was observed on the cartilage surface of the lateral compartment (Fig. 2A). The ACL was partially damaged but maintained tension, whereas the PCL was ruptured and absorbed. Patient's patellofemoral joint was examined and no significant wear was found. The Ligament Advanced Reinforcement System (LARS Company, Arc-sur-Tille, France) was utilized to reconstruct the PCL (Fig. 2B). The femoral tunnel was placed at the junction of the anterolateral bundle and posteromedial bundle at approximately 10:30 of the femoral condyle. Posteromedial and posterolateral knee joint approaches were used, the synovial fat around the PCL stump and the posterior sagittal septum of the knee were appropriately cleaned, and the PCL tibial interdiction and the posterior tibial margin at least 20 mm below it were exposed. The drill guide tibial locator was inserted through the medial patellar approach, crossed over the ACL and circled from the medial PCL stump to the posterior tibial slope to avoid killer turning. The front end of the tibial locator was extended to 20 mm or less below the tibial plateau as much as possible, crossing the PCL's stop point on the original slope to ensure that the adhesion point of the graft after exiting the internal opening of the tibial tunnel was an obtuse angle of the tunnel and reduced ligament wear and cutting. The external opening of the tibial tunnel was made laterally on the tibial crest. After ligament reconstruction, the posterior drawer test yielded negative results, indicating good stability of the knee joint. Subsequently, the patient underwent medial UKA with a St Georg Sled implant (Waldemar Link GmbH, Hamburg, Germany). In order to prevent excessive relaxation of the medial collateral ligament, the midial tibial plateau osteophytes were avoided removing during the procedure. Therefore, sagittal osteotomy appeared to be slightly medial, and part of the tibial plateau prosthesis was ultimately placed on the osteophytes. The flexion–extension gap was checked postoperatively, the flexion and extension functions were restored, and physical examination, such as the drawer test, confirmed that the joint stability was restored. X-ray images taken after surgery showed substantial correction of varus alignment (Fig. 2C, D, and E). No drainage tube was placed postoperatively, resulting in minimal leakage from the incision. The incision exhibited good healing at the 2-week mark, and no complications were observed during the follow-up period.

After surgery, the patient was provided with an adjustable brace for the left knee and encouraged to engage in early rehabilitation training. On the second postoperative day, the patient was allowed to walk with the assistance of crutches. A detailed rehabilitation plan was developed for the patient, which included achieving 120 a degrees of ROM goal by the 3rd week after surgery, continuous muscle strength and gait training, with adjustments based on recovery status and goals. Additionally, early knee straightening and quadriceps muscle strength exercises were recommended. Three weeks after surgery, the patient successfully achieved the rehabilitation goals for range of motion (ROM). The patient was satisfied with the surgical effect. Three months after the operation, the Lysholm score was 81 points, the WOMAC score was 18 points, and the IKDC subjective score was 74.7 points. Infection, poor incision healing, and loosening of the prosthesis were not reported.

## Discussion

In this case report, UKA combined with PCLR was utilized to treat a patient who presented with medial knee OA and PCL deficiency, resulting in positive outcomes during short-term follow-up. This case study may offer valuable treatment insights for individuals with similar conditions.

Clinical studies and treatments for PCL injury and secondary knee medial osteoarthritis are rare [7]. A finite element (FE) study showed that anteroposterior translation significantly increased in patients who underwent UKA for PCL deficiency and high flexion angles and that there was contact stress in the patellofemoral joint and articular cartilage; therefore, the PCL seems to be necessary in UKA [8]. UKA in patients with PCL deficiency may increase the risk of prosthesis wear, loosening, and contralateral compartment OA.

In cruciate ligament reconstruction, the main grafts used are autografts, allografts and synthetic grafts [9]. Autografts, which are typically obtained from tendons such as the quadriceps tendon or the bone-patellar tendon-bone (BPTB), are the most commonly used grafts. The advantages of allograft use include shorter operative times, smaller incisions, and no complications at the donor site. Nevertheless, there are limitations associated with allograft use, including the potential for graft rejection, disease transmission, limited availability, and weakening of the graft structure due to the sterilization process [10, 11]. LARS is a controversial alternative to autografts and allografts as it provides a more robust structure that immediately stabilizes the knee and allows patients to participate in high-intensity rehabilitation sooner; however, there is potential for complications such as rupture, inadequate tendon-bone healing, and loosening [12–14]. Therefore, in order to benefit from quick recovery and reduce the potential occurrence of arthrofibrosis, LARS was chosen as graft substitute in this setting [9].

There are currently two main types of UKA prostheses: mobile-bearing (MB) and fixed-bearing (FB) prostheses. Despite differences in design and mechanics, there are no differences in clinical outcomes, functional results, revision rates, complication rates or relevant scores [15]. Both MB and FB prostheses are used in UKA combined with ACLR. Jaber et al. studied 23 patients who underwent ACLR combined with UKA, all of whom underwent Oxford UKA with autologous hamstring tendons. The functional scores of the patients significantly improved after surgery, and the survival rate was 91.4% at the 14.5-year follow-up [16]. In another study, Foissey et al. performed ACLR combined with robot-guided FB UKA on 10 patients using autologous tendons, and 9 patients returned to sports, but 2 patients underwent arthroscopic release surgery for joint stiffness [17]. In order to avoid the occurrence of bearing dislocation, the FB prosthesis was used. More research is needed to investigate whether the two prostheses differ in this type of surgery.

For patients with significant varus and large osteophytes on the medial side of the knee joint, TKA is the primary surgical option. However, the combination of ACLR and UKA has been shown to be nearly as effective as TKA [16, 18]. Some studies suggest that complete removal of medial osteophytes during UKA may result in MCL relaxation and the potential risk of bearing dislocation. Conversely, retaining osteophytes may not impact surgical outcomes [19]. In this case, the decision was made not to remove medial osteophytes due to concerns about MCL loosening and bearing thickening, and the MCL was appropriately tight for intraoperative exploration. Although this choice caused a medial deviation in the sagittal osteotomy line, resulting in the prosthesis sitting on the osteophytes, the patient did not report any discomfort on the medial side during follow-up.

Postoperative rehabilitation plays a crucial role in restoring knee function and athletic performance. Current studies primarily focus on the postoperative rehabilitation of ACLR, PCLR, and UKA patients. However, there is limited research on postoperative rehabilitation, especially for patients who undergo UKA combined with PCLR. Patients who have undergone artificial ligament surgery can engage in knee flexion and weight-bearing exercises earlier, resulting in a shorter recovery time than that of patients with autografts. Some studies have suggested that patients with artificial ligaments can return to sports activities within 2–6 months after surgery following planned recovery [20]. Relevant studies have shown a significant decrease in quadriceps and hamstring muscle strength after UKA surgery compared to preoperative levels [21]. Therefore, we developed a detailed muscle rehabilitation protocol for this patient, which included early leg straightening combined with quadriceps muscle strength training.

To the best of our knowledge, this is the first reported case of post-PCL-induced medial knee osteoarthritis treated with combined UKA and PCLR. This procedure may be considered appropriate for young patients with unicompartmental knee OA, poor posterior cruciate ligament function, and certain exercise needs. It is crucial to conduct further high-quality clinical research and closely monitor the postoperative outcomes of patients to assess the long-term results and prognosis of this surgical approach.

## Conclusion

This study presents the first reported case of UKA combined with PCLR, demonstrating positive outcomes in the early follow-up period. These findings are evidence of the utility of this novel approach for treating patients with unicompartmental knee OA and concomitant PCL deficiency.

## Data Availability

All data generated or analyzed during this study are included in this published article.
